# Effect of Ar Gas Pressure on LSPR Property of Au Nanoparticles: Comparison of Experimental and Theoretical Studies

**DOI:** 10.3390/nano10061071

**Published:** 2020-05-31

**Authors:** Serap Yiğit Gezgin, Abdullah Kepceoğlu, Yasemin Gündoğdu, Sidiki Zongo, Anna Zawadzka, Hamdi Şükür Kiliç, Bouchta Sahraoui

**Affiliations:** 1 Department of Physics, Faculty of Science, University of Selcuk, Selcuklu 42031, Konya, Turkey; serap3207@hotmail.com (S.Y.G.); abdullahkepceoglu@gmail.com (A.K.); 2 Department of Electric and Energy, Kadınhanı Faik İçil Vocational High School, University of Selçuk, Selçuklu 42031, Konya, Turkey; yasemingundogdu@selcuk.edu.tr; 3 Department of Physics, LPCE, Joseph KI-ZERBO University, 03 P.O. Box 7021, Ouagadougou 03, Burkina Faso; sidiki.zongo@yahoo.fr; 4 Department of Applied Physics, Institute of Physics, Faculty of Physics, Astronomy and Informatics, Nicolaus Copernicus University, Grudziadzka 5, 87-100 Torun, Poland; azawa@fizyka.umk.pl; 5 Directorate of High Technology Research and Application Center, University of Selcuk, Selcuklu 2031, Konya, Turkey; 6 University of Angers, MOLTECH Anjou, CNRS UMR 6200, 2 Bd Lavoisier, F-49045 Angers, France

**Keywords:** gold nanoparticle, thin film, pulsed laser deposition, surface plasmon resonance simulation

## Abstract

In this study, the thin films were produced by using pulsed laser deposition (PLD) technique from gold (Au) nanoparticles deposited on two kinds of substrates under different argon (Ar) gas pressure. Microscope glass slides and silicon (100) wafers were used as amorphous and crystal substrates. The films were deposited under 2 × 10^−3^ mbar, 1 × 10^−2^ mbar, 2 × 10^−2^ mbar argon (Ar) ambient gas pressure. Effect of the background gas pressure on the plasma plume of the ablated Au nanoparticles was investigated in details. Morphology of Au nanoparticle thin films was investigated by means of atomic force microscopy (AFM) technique. Absorption spectra of Au nanoparticles were examined by using UV-Vis spectrometry. Extinction spectra of Au nanoparticles were calculated by using metallic nano particles boundary element method (MNPBEM) simulation programme. Both experimental spectra and simulation data for Au nanoparticles were obtained and compared in this work. It was concluded that they are also in good agreement with literature data. The measurements and the simulation results showed that localized surface plasmon resonance (LSPR) peaks for Au nanoparticles were located in the near infrared region (NIR) because of the larger size of the disk-like shape of Au nanoparticles, and the near-field coupling between Au nanoparticles. It was demonstrated that as the ambient gas (Ar) pressure was increased, the size and the density of Au nanoparticles on the substrate were decreased and the LSPR peak shifts toward the short wavelength region in the spectrum. This shift has been explained by the changes in the morphology of produced thin films.

## 1. Introduction 

Metal nanoparticles with featured size in the range 1–100 nm have unusual chemical and physical properties as compared with its bulk form [1]. When a metal nanoparticle is exposed to electromagnetic radiation, its conduction electrons oscillate collectively through resonant interaction with an incident electromagnetic field. This phenomenon is called as localized surface plasmon resonance (LSPR) effect [2,3,4,5]. Nanostructured materials show interesting optical, electronic, magnetic, and catalytic properties [6], especially. Plasmonic metal nanoparticles containing Au, Ag, and Cu present some spectacular-unique optical properties and these properties are strongly characterized by LSPR effect. Control of the LSPR in metal nanostructures is a very important task for some advanced special applications such as chemical and biological sensing, imaging, plasmon-enhanced detection, photo detection, photo thermal cancer therapy, photovoltaics, optoelectronics, and medicine [7,8]. LSPR strongly depends on the shape, size, density, inter-particle distance, size distribution, and dielectric properties of the nanoparticles as well as substrate or/and surrounding medium [1,9,10]. These parameters affect the movement of conducting electrons within nanoparticles which permit the tuning of the LSPR wavelength region of the nanoparticles [11,12,13].

For the noble metal nanoparticles such as particularly Au and Ag, LSPR band is very strong. The noble metals nanoparticles show absorption peaks in ultraviolet, visible, near infrared, and infrared regions. Au nanoparticles as small as 50 nm exhibit a strong LSPR peak about the wavelength equal to 520 nm. In this work, we showed that LSPR band of Au nanoparticles can be controlled and shifted toward the near infrared (NIR) region of the spectrum. These optical changes were attributed to the morphologic characteristics of nanoparticles such as the comparatively large size, disk-like geometrical shape of Au nanoparticle, and the near-field coupling between Au nanoparticles [5,9,10,12,14,15].

LSPR peaks can be strongly shifted toward the longer wavelengths in the case of following conditions:

- When nanoparticles size is larger than 50 nm, the electrons oscillating in the particle lose energy because of the radiative damping effect and also cannot move coherently because of the phase retardation of the charges. Thus, as the frequency of oscillating electrons decreases, the resonance wavelength increases. [5,16].
- When two or more particles are located close to each other, the electrical fields of the charges collected at the boundaries of each particle increase in the space between the particles [5].
- When the aspect ratio (the ratio of the diameter of nanoparticles to its height) of the disk-like shaped nanoparticles increases, the wavelength increases [10,11,14,17].

Recently, some great efforts have been performed on plasmonic nanoparticles to improve the efficiency of the solar cells. Plasmonic nanoparticles which are embedded in active layers of the thin film solar cells increase the light trapping in the active layer [15]. Therefore, it is possible to obtain semiconductor layer absorbing in the desired region of the solar spectrum by controlling the size of the nanoparticles to adjust the position of LSPR peaks. Thus, this application gives us a strong expectation that high conversion efficiency of photovoltaic devices will be achieved [18].

Characteristic parameters of different coating techniques which are used to grow thin films play an important role in the morphology of thin film. In particular, pulsed laser deposition (PLD) technique has some important flexibility which allows adjusting all parameters of PLD system such as the distance between target and substrate, the laser energy per pulse, focusing conditions of laser beam, laser intensity, substrate temperature, background/ambient gas pressure in the vacuum chamber [19,20]. Moreover, the PLD systems performance is remarkable compared to the other conventional thin film deposition techniques [1,21,22]. In PLD technique, the background/ambient gas pressure in deposition environment is an important parameter that prominently affects the morphology of thin films. The background/ambient gas pressure can be used to adjust the volume of plasma plume expanding into vacuum and the kinetic energy of the ablated particles in the plasma plume, and therefore the particle parameters such as, particle size, number, and size distribution can be controlled by adjusting the ambient gas pressure in the chamber [6,23]. The inert gases like helium (He) and argon (Ar) can shrink the volume of the plasma plume and not react with metal species in the plasma when they are used as an ambient gas. The metal particles in shrunken plasma [6,23,24] collide with each other and the background gas species, and thus, their kinetic energy is reduced. A portion of the ablated particles collide with background/ambient gas species that scatter the particle backward and around, and the number of the ablated particles arriving at the substrate reduce [6,25,26]. It is well-known from the literature that the changes in the morphology of the thin film caused by the background gas pressure affect the optical properties of the thin film [27,28].

In this work, we present important experimental results for the growth mechanism of the Au nanoparticle thin films deposited by PLD at different ambient gas (Ar) pressure. We showed the experimental results of the fabricated nanoparticle thin films by using atomic force microscopy (AFM), scanning electron microscopy (SEM), and UV-vis spectroscopy.

In a nanoparticle, electron density, induced charge transitions, changes in band gap and energy can be determined by many methods. DFT method can be used in determining electronic conditions such as electron density, charge transition excitations, and global potential energy surfaces in nucleated and growing structures [29,30]. Furthermore, MNPBEM toolbox using Maxwell’s equations can be used to calculate external perturbation that induced the electromagnetic fields on the boundaries of plasmonic nanoparticle. It contains plane wave and dipole excitation and the calculation of scattering, absorption, and extinction cross sections. In this study, we present the comparison of the experimentally measured optical properties of Au nanoparticles produced in three different Ar gas pressures and simulation results obtained by using MNPBEM toolbox simulation programme [31,32]. We showed that the experimental results are consistent with the simulation ones.

## 2. Experimental

Gold nanoparticle thin films were deposited by PLD using Nd:YAG laser (Continuum, Minilite II) system which is operated in pulsed mode, emits laser pulses with 5 ns pulse duration and 10 Hz repetition rate at a fundamental wavelength of 1064 nm. The system can also produce second, third, and fourth harmonics of 1064 nm fundamental wavelength at 532 nm, 355 nm, and 266 nm, respectively. We have used the fourth harmonics of the fundamental beam at the wavelength of 266 nm, in this work. Laser pulse power can be controlled by using a neutral density filter and making the power measurements just before the focusing lens.

Both (100)-oriented silicon wafer and glass microscope slide were used as the substrates. The Si wafer was initially cleaned using conventional methods with organic solvents, which were a mixture of 1:1:6 unit of H_2_O_2_ 30%, HCl 37%, and distilled water, respectively, for ten minutes and then rinsed in distilled water for five minutes. The glass microscope slide were first cleaned with soap foam, subsequently was bath in acetone and isopropyl alcohol for fifteen minutes. The cleaning procedure was carried out in an ultrasonic bath, and then substrates were dried in a nitrogen gas flow.

High purity (99.95%, GoodWill Fellow) gold target was used. The substrates and the target were positioned on rotating holders in order to avoid damage to the target surface (for each laser pulse experiences new surface conditions to keep ablation process under homogeneous plasma production) and to produce homogeneous coating. The distance between the target and the substrate was set at 50 mm. All films were deposited on the substrates kept at room temperature (RT). The laser beam energy was equal to 3 mJ per pulse and focused on the Au target surface by a quartz biconvex lens with a focal length of 50 cm. The angle of laser beam’s incidence was equal to 45°. In all experiments, the laser fluence was kept around 23 J/cm^2^. Initially, the background pressure in vacuum chamber before experiment was evacuated down to 5 × 10^−7^ mbar. Afterwards, the gold target was ablated by applying 36,000 laser pulses and the gold thin films were grown in Ar atmosphere at 2 × 10^−3^ mbar, 1 × 10^−2^ mbar, and 2 × 10^−2^ mbar. Morphology of Au nanoparticle thin films deposited on a Si wafer was analyzed by AFM technique. The optical absorption spectra of Au nanoparticle thin films deposited on the microscope slide glass were measured using a UV-Vis spectrophotometer (JASCO, V-670 Spectrophotometer, Waltham, MA, United States).

## 3. Results and Discussion

**Figure 1:**
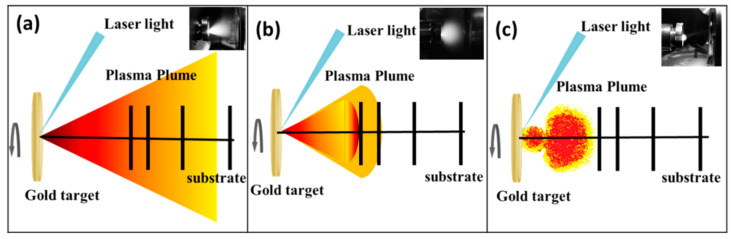
The illustrations of the plasma plume of the ablated Au particles (and pictures in the upper right corner of the illustrations) in Ar gas pressure at (**a**) 2 × 10^−3^ mbar, (**b**) 1 × 10^−2^ mbar, and (**c**) 2 × 10^−2^ mbar, respectively.

In PLD technique the laser ablates target material and produces a plasma plume, which expands freely in vacuum or in diluted background gas ambience (inert gas), as shown in Figure 1a. When the medium is supplied with a gas at low pressure (P ≤ 0.66 mbar), it was observed [25] that the elastic collisions between the ablated particles and the ambient gas atoms have very low probability [25], thus the effect of the ambient gas on the plasma plume can be ignored.

In this study, the Ar ambient gas pressure was held constant at 2 × 10^−3^ mbar which leads to the medium close to vacuum condition and particles dynamics is figured out as in Figure 1a. If the laser ablation is executed in near vacuum conditions (or low ambient gas pressure), the plasma density is higher than that of the background pressure [33]. The ablated particles in the plasma plume which move swiftly in the forward direction and the plasma plume expands because of its high pressure [23]. Although the ablated particles in the plasma plume are subjected to several collisions with each other, and with the gas species in the ambient gas environment, the ablated particles still have enough high kinetic energy and density, and therefore ultimately increase the deposition rate [24]. When ambient Ar gas pressure is increased to 1 × 10^−2^ mbar, the background gas density has a considerable value according to the plasma density. Because of the interaction of the ablated Au particles with ambient Ar gas atoms, the plasma plume propagation slows down and expands, as well as travel length becomes smaller [23] as shown in Figure 1b. It is well-known from the literature [25,34] that the kinetic energy of the ablated particles is decreased because of the larger number of collisions with each other, and Ar background gas species. Therefore, the mean free path of the ablated Au species is reduced [35]. When Ar gas pressure is increased to 2 × 10^−2^ mbar, the more collisions occur because of the ablated Au particle-particle and particle-gas species interactions [35]. Since the plasma plume is under more compression in higher Ar ambient gas pressure (Figure 1c) [34], the ablated particles experience more collisions in path length. Some part of the ablated particles are scattered to around and backwards to the laser irradiated surface [23,25]. Therefore, the deposition rate and the number of particles arriving on the substrate is reduced in this case [36].

**Figure 2:**
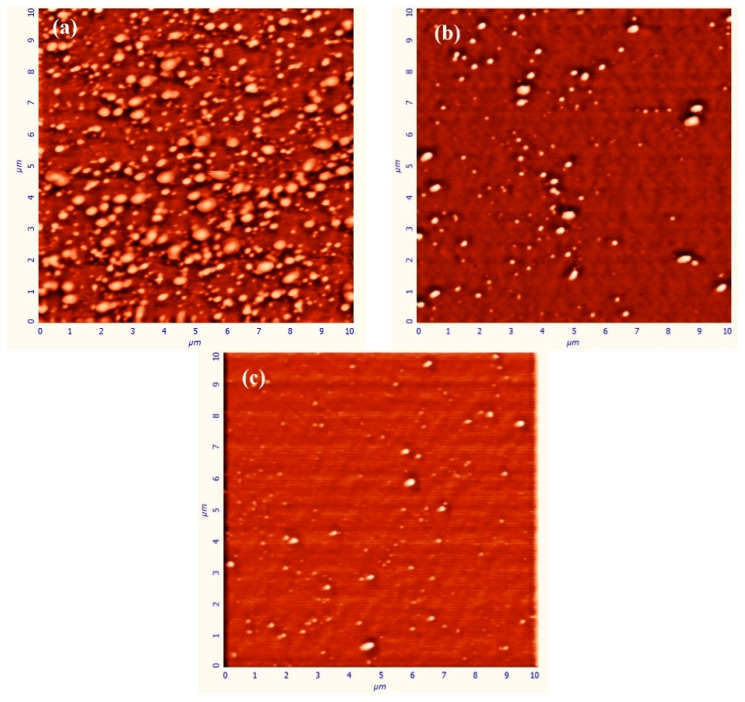
The AFM images of Au thin film deposited in ambient Ar gas pressure at (**a**) 2 × 10^−3^ mbar, (**b**) 1 × 10^−2^ mbar, (**c**) 2 × 10^−2^ mbar, respectively.

It was noticed that the ambient Ar gas plays a very important role in the morphology of the Au nanoparticle thin films, according to the interpretation of results obtained from AFM analysis presented in Figure 2. The ablated Au particles have neither lost significant kinetic energy nor undergone scattering in 2 × 10^−3^ mbar ambient Ar gas pressure. Therefore, the Au particles arriving on Si substrate have enough high energy and diffusion length [37] and, therefore, they tend to form larger nanoparticles by aggregating (Figure 2a) [38]. It can be concluded from the AFM image’s analysis that Au nanoparticles on the substrate have a random distribution in sizes and the average diameter, the inter-particle distance and height for nanoparticles were determined to be 115 nm, 40 nm, and 15 nm, respectively. Au nanoparticles are grown laterally [39] and the shape of Au nanoparticles do not have a perfectly spherical shape. The geometry of the nanoparticles was similar to the disk shape with the flat top. Also, it is acceptable that Au nanoparticle has a large size and are located very close to each other. The near-field interaction occurs between Au nanoparticles, which significantly affects the optical properties of Au nanoparticles. It is also necessary to observe the change of morphological and optical properties by removing the particles from each other. For this, the background (Ar) gas pressure should be increased.

Since the ablated Au nanoparticles in the slightly confining plasma plume collide with each other and the surrounding Ar atoms in 1 × 10^−2^ mbar Ar gas pressure, Au nanoparticles are scattered (around and back) and hence the number of Au nanoparticles reaches on the substrate are reduced [25]. Since ablated Au nanoparticles lost some part of their energy due to the collisions in the plasma plume, the kinetic energy of the Au nanoparticles arriving on the substrate is lower than that in the case of the ambient gas presence at 2 × 10^−3^ mbar [27,34]. Behavior of the particles arriving on the substrate with low kinetic energy (on the substrate) is similar to that of the particles deposited at low substrate temperature conditions [34]. Since the kinetic energy of nanoparticles reaching the substrate is very low, the coalescence of nanoparticles is restricted [40], as seen Figure 2b. Therefore, nanoparticles cannot be grown in larger sizes, the density of the particle is very low since number of particles reaching the substrate which is very low. In this particular case, the average diameter, the inter-particle distance, and height for nanoparticles were determined to be 75 nm, 100 nm, and 13 nm, respectively. In the analysis of the AFM image, the size, inter-particle distance and heights of the nanoparticles were determined by detailed measurements on the two-dimensional structures of the particles. According to literature, the size of particles increases with the increasing background gas pressure [36,37]. But, one of the reasons for the reduction of the particle size with the increasing gas pressure may be that, the ingredient of the plasma may prevent the merger of the particles since the ablated plasma plume has very high temperature and therefore has positively charged particles [41].

When the ambient Ar gas pressure is increased to 2 × 10^−2^ mbar, the collisions between compressed Au particles and ambient Ar gas species become more effective, the velocity of Au nanoparticles decreases faster than that in the case of 1 × 10^−2^ mbar ambient gas pressure and, then, they scatters backwards and around with higher probabilities [42]. Because of the lower velocity and density flow of Au particles at the arrival to the substrate, the coalescence between the Au nanoparticles is not well supplied. Therefore, the grown Au nanoparticles have smaller size and are distributed sparsely, as shown in the Figure 2c. The average diameter, the inter-particle distance, and the height of the Au nanoparticles were determined to be 60 nm, 200 nm, and 11 nm, respectively. In fact, it was expected that the particle size would be even smaller because of the low kinetic energies and densities of these Au nanoparticles, but it has been observed that the particle sizes remain relatively larger than expected because the particles in the shrunken plasma were aggregated together as a result of collision with each other and were cooled by Ar background gas at high pressure [36,37]. As a result, by increasing the Ar gas pressure, the size of the Au nanoparticles reduced and they were separated from each other. As will be described in detail below, the electrical field effect of one nanoparticle on the other nanoparticle is reduced, a more homogeneous and consistent charge distribution can be obtained within Au nanoparticle. The smaller size of the particle contributes to this homogeneous charge distribution. This segregation in the morphological structure will significantly affect the optical properties of Au nanoparticles and will enable these Au nanoparticles to be used in the desired application area depending on the need that needs this optical feature.

**Figure 3:**
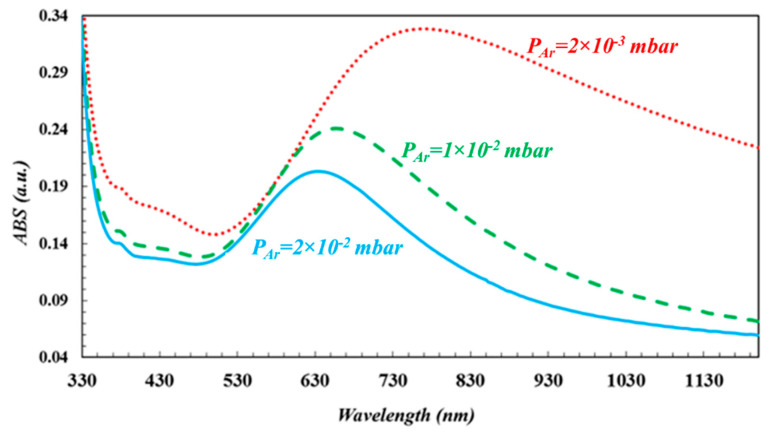
UV-VIS absorption spectra of gold thin films deposited at ambient Argon gas pressures of 2 × 10^−3^ mbar, 1 × 10^−2^ mbar and 2 × 10^−2^ mbar.

The absorption spectra of Au nanoparticles deposited under three different ambient Ar gas pressure conditions are shown in Figure 3. The absorption (LSPR) peaks for the Au nanoparticles appears at the wavelengths equal to 770 nm (red dotted line), 658 nm (green dashed line), and 634 nm (turquoise solid line), respectively. The positions of the LSPR peaks shifted toward the NIR region that have been attributed to three cases which are larger sized Au nanoparticles, the near field interparticle coupling, and the disk-like shaped Au nanoparticles.

The metallic nanoparticles are formed from ionic cores (+) and free electron (−) confined in the nanoparticle. When an electromagnetic field of the light beam impinging on nanoparticles and apply force on conduction electrons, then negative and positive charges are accumulated on both sides of the surface of nanoparticles thus generating an electric dipole. These dipoles create an additional electric field opposite to that which is created by the light beam on the nanoparticle. The coulomb attraction between opposite charges generates a restoring force on the poles of nanoparticles [43]. So, electrons are forced to return to the equilibrium position by this restoring force while electromagnetic field forces them to move away toward the two poles. The restoring force gives rise to these electrons oscillating at a specific frequency [4,44]. Thus, the electrons oscillate with a certain frequency, known as resonance frequency, which is determined by the strength of restoring force [3,45].

The distance between charges located on both sides of the nanoparticle is nearly equal to the diameter of the nanoparticle. As the nanoparticle size is larger than 50 nm, the number of conduction electrons increases and the number of oscillating electrons also becomes larger. However, as the size of nanoparticle increases, the distance between the charges (poles) increases [45] and the interaction between two poles decreases by the phase retardation [16]. Because of this retardation, the restoring force is reduced and the resonance energy decreases and thus, LSPR peak shifts to longer wavelengths (smaller resonant frequency) [4,46]. Also, because of the higher charge densities on the larger nanoparticle, the intensity of LSPR increases. When two nanoparticles are placed next to each other closer than a distance equal or smaller than 2.5 times of the particle diameter, interparticle near field coupling occurs [4,17,47]. Oppositely, the particle behaves as a single-particle dipole mode.

The polarization direction of the incident light has an important role in the near-field coupling between the nanoparticles. So that, the direction of the dipole formed on nanoparticles can be determined by the direction of the incident light [13]. If the incident light has a polarization direction parallel to interparticle axis [5,13,48], the induced dipoles generate local electric fields around the nanoparticle [4,49,50]. If the field applied on nanoparticle is in the same direction as the induced local electric fields, the total electric field experienced by a nanoparticle is the sum of the fields created by the incident light *E_o_* and the near field *E_nf_* of the neighboring nanoparticles [3] and is expressed as:

(1) $$E=E_{o}+E_{nf}$$

The total electric field counteracts to the field of resonance originating from the oscillation of electrons in each Au nanoparticle and hence, weakens the restoring force. As the oscillation frequency of the electrons weakens, the resonance frequency decreases, and the resonance energy increases. Thus, the position of the LSPR peak shifts toward the NIR region [2,5,12,47,48,51,52,53].

The average distances between the couples of nanoparticles presented in Figure 2a–c were determined to be 40 nm, 80 nm, and 200 nm, respectively. Therefore, the interparticle near-field coupling is strong enough for the particles shown in Figure 2a, since particles are located very close to each other. In this case, the system consists of more than two Au nanoparticles, each nanoparticle is exposed to a near field effect applied by other Au nanoparticles, and therefore, this gives rise to a stronger electromagnetic field enhancement. Also, the charge density within nanoparticle is higher in the case when average size of the nanoparticle is about 115 nm in diameter. Therefore, the intensity of LSPR band was higher, and LSPR peak was located in the longer wavelength region. But, when Ar background gas pressure increases, the interparticle distance increases, and therefore, the average size of nanoparticles is reduced as shown in Figure 2c. The field intensity reduces on the surface of nanoparticles, the effect of excitation enhancement decreases [13,54], and thus, the intensity of LSPR peak reduces and then LSPR peak shifts toward shorter wavelength region.

**Figure 4:**
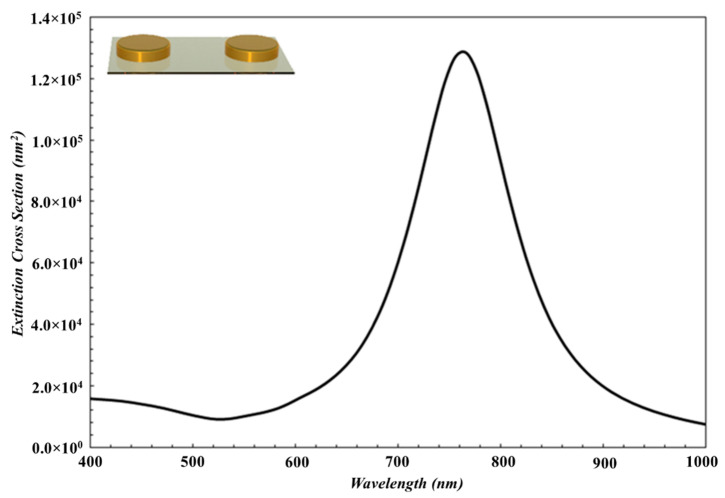
The simulation result of the extinction spectra of disk-like shaped Au nanoparticles with the diameter of 115 nm, height of 15 nm and interparticle distance of 40 nm.

**Figure 5:**
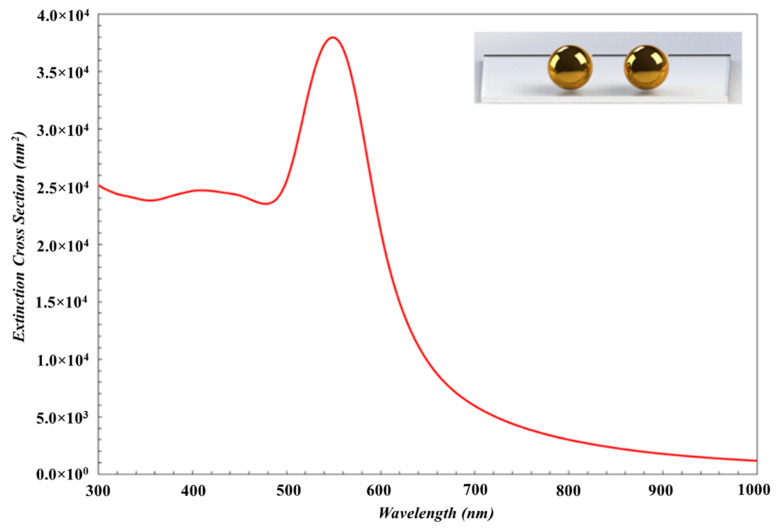
The simulation results of the extinction spectra of the spherical Au nanoparticles with parameters to be 115 nm in diameter and 40 nm for the interparticle distance.

The spectral position of the plasmon resonance of Au nanoparticles also depends on the geometry of particles. In our study, the average diameters of Au nanoparticles deposited on Ar ambient gas at pressures 2 × 10^−3^ mbar, 1 × 10^−2^ mbar and 2 × 10^−2^ mbar were measured to be 115 nm, 75 nm, and 60 nm, while the heights of these Au nanoparticles were measured to be 15 nm, 13 nm, and 11 nm, respectively. According to AFM images, the nanoparticles have larger diameters and lower heights, and have a disk-like shaped structure. In order to show the effect of the particle shape on the LSPR wavelength, LSPR peak of the disk-like shaped Au nanoparticles is red shifted compared to that of spherical nanoparticles as shown in Figure 4 and Figure 5. When the Ar ambient gas pressure is increased, the aspect ratio of disk-like shaped nanoparticles is reduced, and then the LSPR peak shifts toward shorter wavelength region [45].

According to UV-Vis spectrum in Figure 3, the full width at half maximum (FWHM) of LSPR band of Au nanoparticles deposited on Ar gas ambience at 2 × 10^−3^ mbar, 1 × 10^−2^ mbar, 2 × 10^−2^ mbar pressure are 510 ± 3 nm, 210 ± 3 nm, 180 ± 3 nm, respectively. Deposited Au nanoparticles have larger size dispersion on the substrate in the case of 2 × 10^−3^ mbar Ar ambient gas environment. FWHM of LSPR band of the system is the sum of the resonance peaks of the nanoparticles having different sizes [3,46], and therefore, this case causes broader FWHM of LSPR band. In another way, as Ar ambient gas pressure increases, the density and size of Au nanoparticles growing on the substrate are reduced, and then FWHM becomes narrower.

The other reason for a wide LSPR band may be related to higher radiative damping rate which depends on the particle size [5,17]. The radiation damping may be attributed to the energy loss of the electron as a result of photon emission due to movement of conduction electron [2,8]. The elastic scattering of conduction electrons causes dephasing [11], and therefore, the increment of radiation damping implies a dephasing of the coupled plasmons [45]. So, the dephasing is attributed to the loss of resonance energy depending on both larger nanoparticle sizes and interaction between nanoparticles [46]. The larger nanoparticles are formed by aggregation of smaller nanoparticles wherein the density of free electrons is higher [55]. As the nanoparticle size increases, the radiative damping rate increases, and the collective electron oscillations undergoes the time retardation depending on the electron density. Therefore, LSPR energy decreases and the wavelength of LSPR redshifted [55].

### 3.1. Simulation Results

In this study, we have carried out MNPBEM simulation studies for nanoparticles and compared them to the experimental results. MNPBEM is the simulation toolbox for metallic nanoparticles, which is established on a boundary element method. It contains plane wave and dipole excitation and the calculation of scattering, absorption, and extinction cross sections. MNPBEM toolbox, works in basis for dielectric environment where bodies in homogeneous and isotropic dielectric functions are discretized by interfaces. It can only separate the boundaries between different dielectric materials, not the entire volume. Gauss units are used in the toolbox, which is beneficial for vector and scalar potentials [31,32].

#### Theory

As stated earlier, when a nanoparticle is excited by external perturbation, such as the electrical field of incoming light or the electrical field formed by the neighboring nanoparticle, it is polarized and the electromagnetic field is induced. MNPBEM toolbox aims to calculate the external perturbation that induced this electromagnetic field. This computation is carried out by the solution of Maxwell’s equations and the use of boundary conditions at nanoparticle boundaries. The dielectric nanoparticles are defined by local and dielectric functions *ε_j_*(*ω*), which are discretized by clear boundaries ∂*V_j_*. The magnetic permeability is set to µ = 1, taking into account the Maxwell’s equations in ω of frequency space. The main contents of BEM approach are *ϕ*(*r*) scalar and *A*(*r*) vector potentials, are connected to electromagnetic fields through the Equation (2)

(2) E=ikA−∇ϕ, B=∇×A

where *k = ω/c* is the wavenumber and *c* is the light speed in vacuum. The potentials are related by Lorentz gauge condition ∇*A = ikεϕ*. In each medium, Green function is introduced to Helmholtz equation described via

(3) $$\left(\nabla^{2}+k_{j}^{2}\right)G_{j}\left(r,r^{\prime }\right)=-4\pi \delta \left(r-r^{\prime }\right),\;G_{j}\left(r,r^{\prime }\right)=\frac{e^{ik_{j}\left|r-r^{\prime }\right|}}{\left|r-r^{\prime }\right|},$$

here, $k_{j}=\sqrt{\varepsilon_{j}}k$ is the wavenumber in medium *r* ∈ *V_j_*. To heterogeneous dielectric environment, the solutions of Maxwell’s equation are given below:

(4) $$\phi_{j}\left(r\right)=\phi_{j}^{e}\left(r\right)+\oint_{\partial V_{j}}G_{j}\left(r,s\right)\sigma_{j}\left(s\right)da$$

(5) $$A_{j}\left(r\right)=A_{j}^{e}+\oint_{\partial V_{j}}G_{j}\left(r,s\right)h_{j}\left(s\right)da$$

here, $\phi_{j}^{e}$ and $A_{j}^{e}$ are scalar and vector potentials, which qualified the external perturbation in a given medium *j*. *σ_j_* is the surface charge and ***h****_j_* is current distributions, which are selected such that boundary conditions of Maxwell’s equations at the interfaces between the regions have the different permittivities ((*ε_j_* for *ε*_1_—particle inside; *ε*_2_—particle outside).

With MNBEM toolbox based on the equations above, to calculate the extinction cross section of nanoparticle placed directly on the substrate; the dielectric coefficient of the medium and the metal nanoparticle, the refractive index of the substrate, the shape, dimensions, and height of the metal nanoparticle and the distances between the nanoparticles are entered in MNBEM toolbox by commanding. As a result of running the program, the extinction cross section spectra is obtained [31,32].

In this study, simulation studies were performed by using an Intel Core i7-4510U 3.10 GHz 64-bit computer with 8 GB of RAM (1600 MHz) for the calculations. The CPU time under Windows 8.1 operating system was approximately 3 h for each extinction spectrum of the two disk-shaped nanoparticles. In this study, the data entered into MNBEM toolbox for disc-like shaped Au nanoparticles deposited on glass substrate is: the dielectric coefficient of air and gold of nanoparticle are 1 and 2.5, the refractive index of glass substrate is 1.52 [31,32]. The diameter, the inter-particle distance and height of disk-like shaped Au nanoparticles grown in Ar gas ambience at 2 × 10^−3^ mbar; 1 × 10^−2^ mbar; 2 × 10^−2^ mbar pressure, which are 115 nm, 40 nm, 15 nm (for 2 × 10^−3^ mbar pressure); 75 nm, 100 nm, 13 nm (for 1 × 10^−2^ mbar pressure) and 60 nm, 200 nm, 11 nm (for 2 × 10^−2^ mbar pressure), respectively.

First, in order to determine the shape of nanoparticles specifically, the simulation results were compared with the experimental results for the spherical and disk-like shape nanoparticles. Also, extinction spectra for nanoparticles were obtained by using MNPBEM simulation programme depending on the near field coupling between disk-like nanoparticles and the near field coupling between spherical nanoparticles. It was observed that, the experimental data fits the theoretical results for the disk-like nanoparticles. The extinction simulation spectrum of disk-like shape Au nanoparticles is displayed in Figure 4 and it was noticed that these theoretical results are very similar to that deposited experimentally at 2 × 10^−3^ mbar Ar ambient gas pressure. The diameter, height, and the interparticle distances of disk-like shaped Au nanoparticles are determined to be 115 nm, 15 nm, and 40 nm, respectively.

Theoretical background for MNPBEM implies that the extinction efficiency is inversely proportional to the distance between Au nanoparticles. Since the disk-like shape nanoparticles have the larger size and the near field interparticle coupling, LSPR peak have consequently shifted toward the IR region. Also, theoretical extinction spectrum was compatible with the experimental results obtained in present work. The widths of the experimental LSPR bands are greater than those for theoretically obtained results. The reason for this is that Au nanoparticle thin film grown on the substrate has high particle density, the greater size distribution and different sizes as indicated in Figure 2a. At the simulation program, it has been considered that the nanoparticles are smooth three-dimensional geometrical objects with exact sizes and predefined interparticle distances. Therefore, simulated LSPR band is more uniform and narrower. Although LSPR band in the simulation is shifted a few nm because of the consideration of experimental result, both results are in well agreement (LSPR peak is presented with red dot line in Figure 3).

The diameter and interparticle distances for spherical nanoparticles were taken to be 115 nm and 40 nm in average (Figure 2a), respectively, measured by AFM. The extinction spectrum was obtained by using MNPBEM simulation program is presented in Figure 5. Because of authors’ knowledge, the multipoles are induced on the individual particles due to the near field coupling between the larger spherical nanoparticle. Also, the larger sized nanoparticles lead to higher modes for the electrons confined in nanoparticles (nanoparticle size ≥ 100 nm) [2] and no homogenous electric field is created, and therefore, electron cloud not move homogeneously. The charge distribution on nanoparticle surface is inhomogeneous and the high multipolar charge distribution is excited [2]. Therefore, two peaks are formed in an extinction spectrum and LSPR peak obtained from simulation is located at a wavelength that is shorter than what we have obtained from the experimental work. Consequently, the simulation results are not in agreement with experimental result (LSPR band with red dot line in Figure 3). This result is concluded that Au nanoparticles in this study are not spherical.

**Figure 6:**
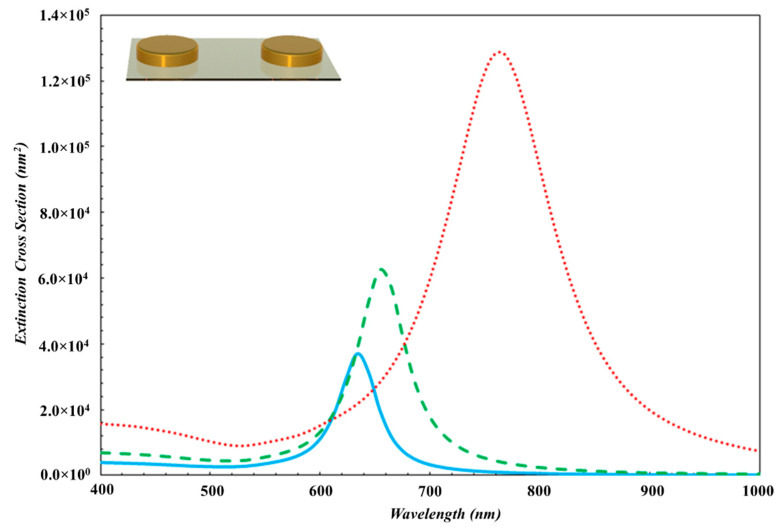
The simulation results of the extinction spectra of Au nanoparticles deposited at 2 × 10^−3^ mbar, 1 × 10^−2^ mbar and 2 × 10^−2^ mbar of Ar gas pressure.

Experimental and theoretical results obtained in this work are given in Figure 3 and Figure 6, respectively. From the comparison of the both spectra, one can see that the LSPR bands obtained by both simulation and experimental studies are consistent even if the LSPR bands shifted a few nm in the theoretical one, as seen in Table 1. When the ambient gas pressure was increased, the average diameter and the height of the disk-like shaped Au nanoparticles became smaller while the inter-particle distance became larger. Therefore, the near field inter-particle coupling became weaker. The electromagnetic field enhancement in the gap between nanoparticles was reduced. It has been observed that as the size of Au nanoparticles decreases, the radiative damping rate decreases too. If we consider it in terms of theoretical results, MNPBEM toolbox determines the position of the LSPR peak for the external perturbation induced electromagnetic fields, using boundary conditions at Au nanoparticle boundaries. With the reduction of Au nanoparticle size, surface charge and current distributions change at the interface at the particle boundary. This change significantly affects the electric field of the oscillating dipole [31,32]. Thus, LSPR peak is shifted toward shorter wavelength region and the measured intensity of LSPR peak is reduced.

## 4. Conclusions

It is well-known that the active layer of the most solar cells has a weak absorption in the longer wavelength region, and therefore, the power conversion efficiency of the solar cell remains limited. We have carried out this work to get an answer for the question of whether we can improve the conversion efficiency of solar cells by enhancing the absorption rate of light in the longer wavelength region by embedding Au nanoparticles in the active layer.

In this study, Au nanoparticle thin films have been deposited by using PLD system at different Ar ambient gas pressures and the tunability of LSPR peak position has been described. LSPR peaks of Au nanoparticles grown in Ar gas ambience at 2 × 10^−3^ mbar, 1 × 10^−2^ mbar, 2 × 10^−2^ mbar pressure, were located on 770 nm, 658 nm, and 634 nm in solar spectrum, respectively. LSPR peak of the Au nanoparticle system was located in the long wavelength region. We observed that PLD-produced particles have been grown laterally, and they resembled a disk-like shape. Therefore, LSPR peak at long wavelengths was attributed to the larger size of disk-like shaped Au nanoparticles and, in addition, to the near field interparticle coupling. According to several investigations found in the literature, the LSPR peaks of the PLD-grown Au plasmonic nanoparticles are generally located in the NIR region of the spectrum. However, the red shift in the LSPR peak of Au nanoparticles is not clearly expressed in the literature. With this study, we have emphasized that the red shift of the LSPR peak of Au nanoparticle thin films is based on the lateral growth of disc like structure of Au nanoparticles produced by PLD. These results are strongly supported by theoretical simulation data obtained by running MNPBEM simulation programme. The position of the LSPR peaks of the PLD-grown Au nanoparticle thin films appears close to the NIR region without a special process that is a great advantage for applications in photovoltaic, medicine, and sensing. In addition, Ar gas pressure was used to shift the basic LSPR peak position of the gold nanoparticles. As the ambient gas pressure was increased, the size of Au nanoparticles became smaller and the interparticle distance became larger, as a consequence of that, LSPR peak was shifted toward the shorter wavelength region. For Ar gas pressure at 2 × 10^−2^ mbar, 1 × 10^−2^ mbar, and 2 × 10^−3^ mbar, LSPR peak of disc-like Au nanoparticles have been calculated to be 637 nm, 656 nm, and 764 nm by MNPBEM toolbox, respectively. In order to prove that the long wavelength positioned LSPR peak is due to large disc-like Au nanoparticles, the extinction spectrum was obtained by performing MNPBEM simulation for both the disk-like and the spherical shape nanoparticles and the simulation results obtained for disk-like shaped particle is in agreement with the experimental absorption spectra while that disagreed for the spherical shaped nanoparticles. Also, LSPR peaks of the nanoparticles grown at higher Ar ambient gas pressures are in close proximity with simulation results.

## Figures and Tables

**Table 1:** The experimental and theoretical results of Au nanoparticles grown in Ar gas pressure at 2 × 10^−3^ mbar, 1 × 10^−2^ mbar, 2 × 10^−2^ mbar pressure.

Ar gas Pressure	Particle Size; Inter-Particle Distance; Height	Experimental LSPR Peak Wavelengths	Theoretical LSPR Peak Wavelengths
2 × 10^−3^ mbar	115 nm; 40 nm; 15 nm	770 nm	764 nm
1 × 10^−2^ mbar	75 nm; 100 nm; 13 nm	658 nm	656 nm
2 × 10^−2^ mbar	60 nm; 200 nm; 11 nm	634 nm	637 nm
